# Akt: a key transducer in cancer

**DOI:** 10.1186/s12929-022-00860-9

**Published:** 2022-10-01

**Authors:** Pei-Jane Tsai, Yi-Hsin Lai, Rajesh Kumar Manne, Yau-Sheng Tsai, Dos Sarbassov, Hui-Kuan Lin

**Affiliations:** 1 Department of Cancer Biology, Wake Forest University School of Medicine, Winston-Salem, NC 27157 USA; 2 Department of Medical Laboratory Science and Biotechnology, College of Medicine, National Cheng Kung University, Tainan, Taiwan; 3 Institute of Basic Medical Sciences, College of Medicine, National Cheng Kung University, Tainan, Taiwan; 4 Institute of Clinical Medicine, College of Medicine, National Cheng Kung University, Tainan, Taiwan; 5 Biology Department, School of Sciences and Humanities, and National Laboratory Astana, Nazarbayev University, Nur-Sultan City, 010000 Kazakhstan

**Keywords:** PI3K, Akt, TRAF6, Skp2, Cancer, Posttranslational modifications

## Abstract

Growth factor signaling plays a pivotal role in diverse biological functions, such as cell growth, apoptosis, senescence, and migration and its deregulation has been linked to various human diseases. Akt kinase is a central player transmitting extracellular clues to various cellular compartments, in turn executing these biological processes. Since the discovery of Akt three decades ago, the tremendous progress towards identifying its upstream regulators and downstream effectors and its roles in cancer has been made, offering novel paradigms and therapeutic strategies for targeting human diseases and cancers with deregulated Akt activation. Unraveling the molecular mechanisms for Akt signaling networks paves the way for developing selective inhibitors targeting Akt and its signaling regulation for the management of human diseases including cancer.

## Introduction

Cells respond to various extracellular clues, such as growth factors and cytokines for their proliferation and survival by engaging their cognate receptors. The PI3K (phosphatidylinositol 3-kinase)/Akt pathway is an important pathway that transmits these stimuli from outside to the nucleus inside of the cells. Deregulation of this pathway is associated with numerous types of human diseases, such as diabetes and cancers [1, 2]. Because of its important role in cell signaling, the PI3K/Akt pathway has become one of the most intensely studied areas in the last decades.

PI3Ks are divided into three classes: **Class I**, **Class II**, and **Class III**. Among them, Class I PI3K is the most classical one that can be further divided into the Class IA and IB [1, 2]. Class IA of the PI3K consists of the p85 regulatory subunit and p110 catalytic subunit that together form a heterodimer. By binding to the p110 catalytic subunit, the p85 regulatory subunit keeps PI3K in an inactive state until stimulated by growth factors or cytokines. In response to these physiological cues, the p85 subunit of PI3K is phosphorylated and relieves its inhibition on PI3K for its activation. The activated PI3K then triggers the formation of PIP3 (phosphoinositol 3, 4, 5-triphosphate) in the plasma membrane by phosphorylating PIP2 (phosphoinositol 4, 5-biphosphate), a second messenger that interacts with the PH domain of Akt and recruits Akt to the plasma membrane for activation [1, 2].

Akt known also as protein kinase B (PKB) is a serine-threonine protein kinase consisting of three isoforms, Akt1, Akt2, and Akt3, which are encoded by three distinct genes *PKBα*, *PKBβ,* and *PKBγ*, respectively [3, 4]. Akt contains the N-terminal **pleckstrin homology (PH) domain**, central catalytic domain and C-terminal regulatory region. The PH domain is critical for Akt membrane recruitment, whereas the central catalytic domain and C-terminal regulatory region are required for Akt kinase activation [3, 4]. Genetic evidence suggests that these isoforms display distinct biological functions although the underlying mechanism responsible for these differences remains unclear. Although *Akt1* null mice display no obvious developmental abnormality, their body weights are significantly reduced, indicating a critical role for Akt1 in cell survival. *Akt2* null mice develop severe type 2 diabetes, suggesting a central role for Akt2 in the maintenance of glucose homeostasis. While *Akt3* null mice show impaired brain development, indicating a role for Akt3 in brain development [5]. Of note, overexpression of specific Akt isoforms has been found in different cancers, such as *Akt1* amplification has been reported in breast, gastric cancers, *Akt2* in hepatocellular carcinomas, colorectal, ovarian and pancreatic cancers, and *Akt3* amplification in estrogen receptor-negative breast tumors and melanoma [6]. In addition, E17K hotspot is the most characteristic mutation of the Akt1 which is a recurrent somatic mutation observed in breast cancer, colorectal cancer, lung cancer and ovarian cancer. Similar mutation in Akt2 and Akt3 was rare [7]. For the role of Akt isoforms in cancer development, earlier studies revealed that systematic *Akt1* deficiency inhibited primary cancer development, but promotes breast cancer migration and metastasis [8, 9]. In contrast, systematic *Akt2* loss promoted breast cancer development but impairs breast cancer migration and metastasis [8, 9], indicating distinct functions of Akt isoforms in breast cancer development, breast cancer migration and metastasis. However, recent study demonstrated that both breast-specific Akt1 and Akt2 deletion impaired breast cancer development driven by ErbB2 overexpression, but breast cancer metastasis was not affected by breast-specific *Akt1* deletion [10]. Thus, the systematic versus cell-autonomous deletion of *Akt *isoforms could result in distinct impacts on cancer phenotypes.

In this review, we summarized the recent provocative findings on Akt regulations and their roles in signaling and cancer control as well as therapeutical implications. Unraveling the molecular mechanisms for Akt signaling regulation paves the way for developing promising targeting strategies and agents for the management of human diseases including cancer.

## Posttranslational regulation of Akt

**Fig. 1:**
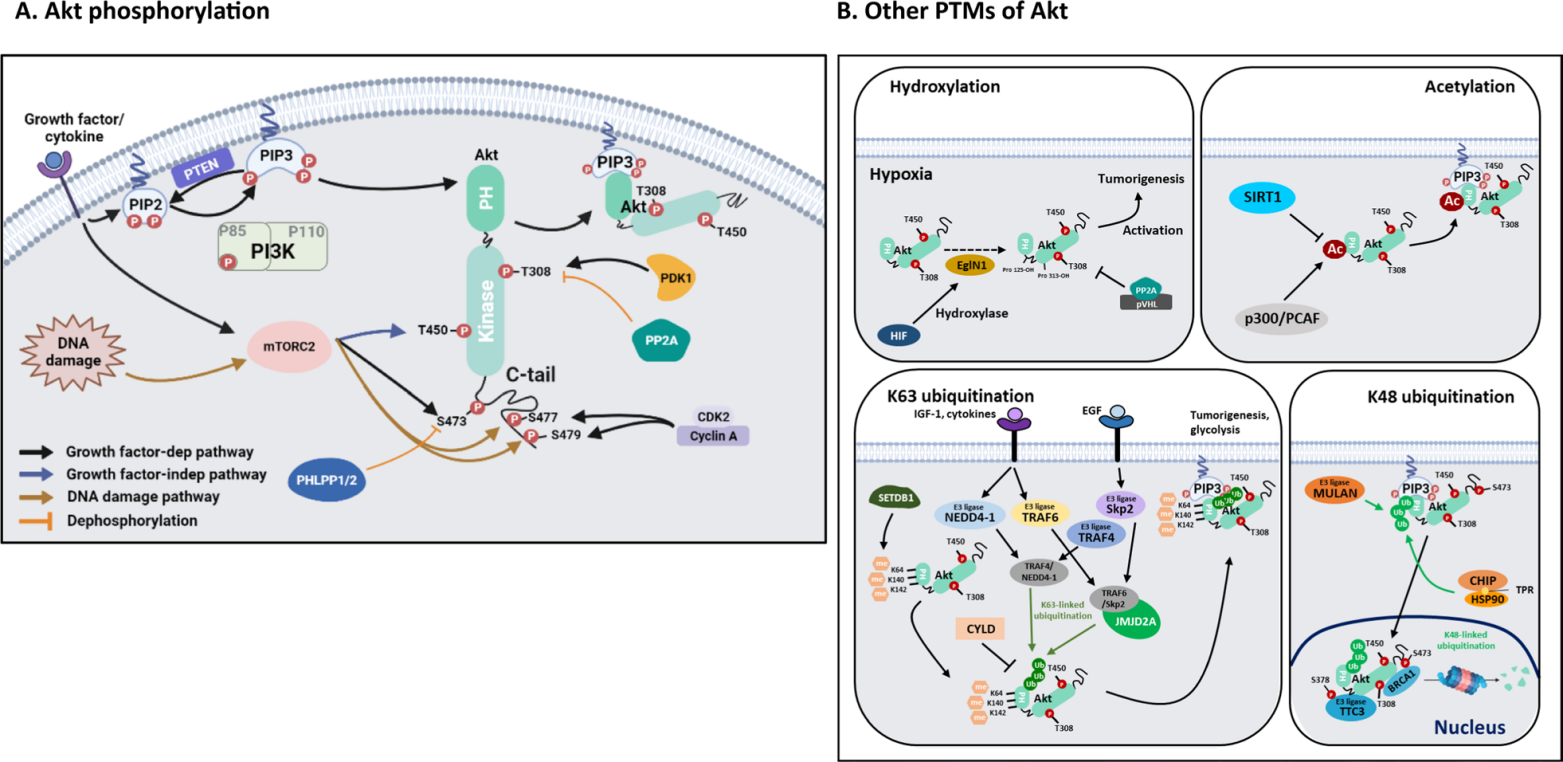
Diverse posttranslational modifications (PTMs) of Akt regulate Akt signaling activation and inhibition. **A** Phosphorylation of Akt. **B** Other PTMs of Akt, including hydroxylation, acetylation, methylation, and ubiquitination

Phosphorylated Akt is crucial for Akt kinase activation after membrane translocation. Besides, several recent studies revealed that other Akt posttranslational modifications (PTMs), such as ubiquitination, acetylation and hydroxylation, have also been reported to alter Akt kinase activity and/or activation [11–14]. We summarized the regulation of Akt PTMs and its role in Akt signaling activation in the following section (Fig. 1).

### Akt phosphorylation

After PI3K/Akt pathway is activated by numerous growth factors and cytokines, human Akt1 is recruited to the plasma membrane [1, 2] and phosphorylated by PDK1 at T308 within its catalytic domain, which is critical for Akt kinase activation. Human Akt1 is also phosphorylated by a protein complex formed by serine/threonine kinase **mTORC2** (mTOR complex 2), consisting of mammalian target of rapamycin (mTOR), mammalian stress-activated protein kinase interacting protein 1 (mSIN1), rapamycin-insensitive companion of mTOR (RICTOR), and mammalian lethal with sec-13 protein 8 (mLST8), at S473 located in the C-terminal regulatory region either in plasma membrane or endoplasmic reticulum (ER) [15]. Phosphorylation of Akt at both residues leads to its full activation [16, 17]. However, phosphorylation of human Akt1 at T450 by mTORC2 maintains its activation in a growth factor-independent manner [18]. Interestingly, **CDK2** (Cyclin dependent kinase 2)/**Cyclin A**, an protein kinases characterized by cyclin that provides domains essential for its enzymatic activity, phosphorylates Akt at S477 and T479 located on C-terminal regulatory region to optimize its C-tail confirmation leading to facilitating Akt S473 phosphorylation and enhancing Akt kinase activity in a cell cycle-dependent manner [19]. Moreover, this phosphorylation can be also induced by DNA damage and growth factor signaling through mTORC2 activation [19].

### Akt dephosphorylation

In contrast, **PTEN** (Phosphatase and tensin homolog) tumor suppressor commonly mutated in many human cancers displays phosphatase activity by removing one phosphate from **PIP3** and in turn inactivating Akt [20, 21]. Thus, PTEN is a negative regulator for PI3K/Akt signal. Apart from signal termination by PTEN lipid phosphatase, two critical protein phosphatases function to directly inactivate Akt (Fig. 1). **Protein phosphatase 2A** (PP2A) dephosphorylates Akt at T308, leading to its kinase inactivation [22]. The PP2A B55α regulatory subunit can directly bind to Akt in lymphoid cells [23], whereas the B56β subunit directs PP2A to Akt in adipocytes [24]. The **PH domain leucine-rich repeat protein phosphatases** (PHLPP1 and PHLPP2) were discovered as the physiological Akt S473 phosphatases [25]. PHLPP1 and PHLPP2 dephosphorylate S473 on specific AKT isoforms [26]. Since loss of PHLPP activity leads to hyperphosphorylation of Akt, it is not surprising that PHLPP1/2 expression is reduced or lost in many cancers [27].

### Akt hydroxylation

**Egl-9 family hypoxia inducible factor 1** (**EglN1**) is a proline hydroxylase acting as an oxygen sensor that regulates the degradation of hypoxia-inducible factor α (HIFα). Under normal oxygen concentration, EglN1 hydroxylates Akt1 on Pro125/Pro313 and promotes the binding of pVHL, in turn leading to inhibiting Akt activity through ubiquitination manner [28, 29]. Under hypoxic conditions, **EglN1** is incapable of hydroxylating Akt, which facilitates **pVHL**-mediated enrollment of PP2A to dephosphorylate Akt-T308, thereby leading to activation of Akt [12]. This discovery of Akt hyperactivation by hypoxia offers a potential mechanism by which hypoxic environment habitually come across in solid tumors to facilitate tumorigenesis and drug resistance.

### Akt acetylation

The PH domain of Akt plays a significantly regulatory role to maintain its inactive and active state by changing its confirmation before and after binding to the plasma membrane [30]. It is conceivable that PTMs occurring on the Akt PH domain may impact the membrane translocation of Akt to accomplish its activation and signaling regulations. In support of this notion, **p300/PCAF** has been shown to promote Akt membrane translocation and kinase activity by acetylating Akt at its PH domain, which is abrogated by **SIRT1** deacetylase [14]. Collectively, acetylation of Akt on its PH domain serves a barrier for Akt activation.

### Akt K63-linked ubiquitination

Akt membrane translocation has been characterized as a key event for Akt activation. This event is critically maintained by lysine (K)-63-linked ubiquitination of Akt, in addition to classical PI3K activation. Upon growth factor or cytokine stimulation, Akt undergoes non-proteolytic K63-linked ubiquitination on K8/K14 residues within its PH domain to promote membrane translocation and subsequent phosphorylation and activation of Akt. The first identified E3 ligase for K63-linked ubiquitination of Akt in response to the stimulation of **IGF-1** (insulin-like growth factor-1, a hormone that manages the effects of growth hormone) or cytokines was **TRAF6** (TNF receptor-associated factor 6) [4, 13, 31]. Loss of TRAF6 impairs K63-linked ubiquitination, membrane recruitment and activation of Akt, resulting in reduced prostate tumor growth, making it a promising novel anti-cancer target. NEDD4-1 was characterized as an additional E3 ligase that could drive K63-linked ubiquitination of Akt for Akt activation upon IGF-1 stimulation [32]. While IGF-1 can initiate the Akt K63-linked ubiquitination, EGF was also identified to be responsible for this event by utilizing Skp2 (S-phase kinase-associated protein-2) and **TRAF4** E3 ligases [33, 34]. Skp2 deficiency impairs Akt ubiquitination and activation, resulting in defective glycolysis and tumorigenesis. Of note, EGF stimulation selectively enhances the interaction of Akt with Skp2 and Skp2 E3 ligase activity, but not with TRAF6 and TRAF6 E3 ligase activity. In contrast, while IGF-1 stimulates TRAF6 activity within 15 min, it does not induce Skp2 activation at this early time point. These findings collectively postulate a fascinating model that although Akt K63-linked ubiquitination is a common event to drive Akt activation, distinct extracellular cues engaged in this process selectively utilize different E3 ligases. Thus, both K63-linked ubiquitination of Akt and PIP3/Akt binding are important events required for Akt membrane recruitment and activation. It is important to note that although K63-linked ubiquitination of Akt occurs before PIP3 binding, it does not affect the PIP3 binding and vice versa. Similar to phosphorylation, ubiquitination is also a reversible process that can be removed by deubiquitinating enzymes. In the case of Akt deubiquitination, **CYLD** is the deubiquitinase responsible for removing ubiquitin chain of Akt. The loss of CYLD promotes Akt hyperubiquitination and activation, as well as cell proliferation, survival and prostate tumorigenesis [35, 36]. Accordingly, CYLD tumor suppressor is a negative regulator for Akt activation by removing K63-linked ubiquitination of Akt, although the identity of other Akt deubiquitinase remains to be discovered.

### Akt methylation

Given the key role of K63-linked ubiquitination of Akt in Akt membrane recruitment and activation, understanding the regulatory mechanisms by which E3 ligases sense growth factor stimulation for Akt ubiquitination and activation is of significance. We showed that the K63-linked ubiquitination of Akt is facilitated by Akt K64 methylation, which serves a key upstream signal to enable the interaction of Akt and its E3 ligases essential for K63-linked ubiquitination, membrane localization and activation of Akt upon growth factor stimulation [37]. **SETDB1** (SET domain bifurcated histone lysine methyltransferase 1) is identified as a methyltransferase that induces Akt tri-methylation at K64, which is recognized by **JMJD2A** that interacts with E3 ligases such as TRAF6 and Skp2 and recruits them to Akt for eliciting K63-linked ubiquitination and activation of Akt in response to growth factor stimulation [37]. This study underscores the key role of SETDB1-mediated Akt K64 methylation, which serves as a poor prognosis marker in non-small cell lung cancer (**NSCLC**) patients, in driving Akt ubiquitination and hyperactivation for promoting cancer progression, hence representing a novel paradigm for targeting **NSCLC**. In support of these findings, Wei’s group also revealed that SETDB1-mediated Akt methylation at K140/K142 plays a significant role in the interaction of Akt with its E3 ubiquitin ligase to facilitate its membrane translocation and kinase activation [38]. Therefore, development of a specific inhibitor against SETDB1 may serve as a promising agent for targeting Akt-driven human cancers.

### Akt K48-linked ubiquitination

On the other hand, Akt undergoes K48-linked ubiquitination resulting in its degradation (Fig. 1). An earlier study reported that **TTC3** (tetratricopeptide repeat domain 3) is an E3 ligase responsible for Akt ubiquitination and degradation [39]. Several lines of evidence using in vitro and in vivo experiments revealed that Akt induces phosphorylation of TTC3 at Ser378, and such phosphorylation is required for TTC3 E3 ligase activity, which in turn drives K48-linked ubiquitination and degradation of Akt, thereby offering a negative feedback loop mechanism for Akt stability [39]. Of note, BRCA1 (breast cancer susceptibility gene 1), a tumor suppressor with E3 ligase activity, interacted with phosphorylated Akt at T308 and S473 and triggered K48-linked ubiquitination and proteasome degradation of Akt especially in nucleus [40]. In cytosol, phosphorylated form Akt was also ubiquitinated and negatively regulated by E3 ligase **MULAN** [41]. However, this study does not distinguish the specificity of ubiquitination between T308 and S473 of Akt by MULAN. Furthermore, **CHIP** (chaperone-associated ubiquitin ligase) was reported to interact with **HSP90**, which stabilizes Akt [42], through its TPR (tetratricopeptide repeat) motif, thereby orchestrating Akt ubiquitination. These studies indicate that K48-linked ubiquitination of Akt occurs in cytosol by MULAN and CHIP, whereas it appears in nucleus by BRCA1 and TTC3 [39–42]. Thus, K48-linked ubiquitination of Akt by its various E3 ligases may differentially affect its cytosolic and nuclear substrates, thereby coordinating to regulate diverse cellular responses. However, how these E3 ubiquitin ligases distinctively orchestrate Akt is currently mysterious. Further studies will be required to further resolve this puzzle.

### Akt SUMOylation

SUMOylation is an enzymatic cascade reaction catalyzed by covalently conjugating small ubiquitin-related modifiers (SUMO) to a lysine residue in target proteins via its carboxyl-terminal glycine in the processed SUMO [43]. There are 34 lysine residues in Akt protein sequence. SUMOylated Akt was found to regulate cell proliferation, cell cycle and tumorigenesis [44, 45]. Akt SUMOylation is enhanced under both physiological and pathological conditions, including the stimulation of insulin, IGF-1 and heat-shock [46]. The major acceptor site for SUMOylation Akt through SUMO1 conjugation is located at K276 promoted by SUMO E3 ligase PIAS1  (Protein Inhibitor Of Activated STAT 1) and reversed by SENP1 (SUMO1/sentrin specific peptidase 1) [44]. Akt SUMOylation in a manner dependent on Akt phosphorylation increases Akt kinase activity without impacting on the phosphorylation level of Akt [46]. Akt directly phosphorylates Ubc9 and SUMO1 at Thr35 and Thr76, respectively. Phosphorylated Ubc9 and SUMO1 regulates global SUMOylation, including STAT1, CREB (cAMP-response element binding) and PTEN SUMOylation [46]. SUMOylated Akt mainly localized in the nucleus increases cyclin D1 expression and cell proliferation. In addition, the cancer derived mutant E17K in Akt1 was more efficiently SUMOylated than wild-type Akt1. Loss of SUMOylation on Akt1 E17K reduced cell proliferation, cell migration and tumorigenesis [46].

### Akt glycosylation

In addition, Akt also undergoes *O*-linked *N*-acetylglucosamine glycosylations (*O*-GlcNAcylation), which is a dynamic and reversible modification on serine and threonine residues leading to interfering the cellular signaling and function. Interestingly, *O*-GlcNAcylation of Akt at T305 and T312 inhibits Akt phosphorylation at T308 via disrupting the interaction between Akt and PDK1 [47]. As a result, this modification causes the suppression of tumor cell proliferation and migration. However, O-GlcNAcylation of Akt at T430 and T479 promotes Akt phosphorylation on S473 and activation by reducing the binding of Akt with mTORC2, thus resulting in blocking vascular smooth muscle cell calcification [48]. Future study is warranted to further dissect the role of this modification and its mechanisms in cancer progression.

## Akt signaling orchestrates cellular functions and tumorigenesis

**Fig. 2:**
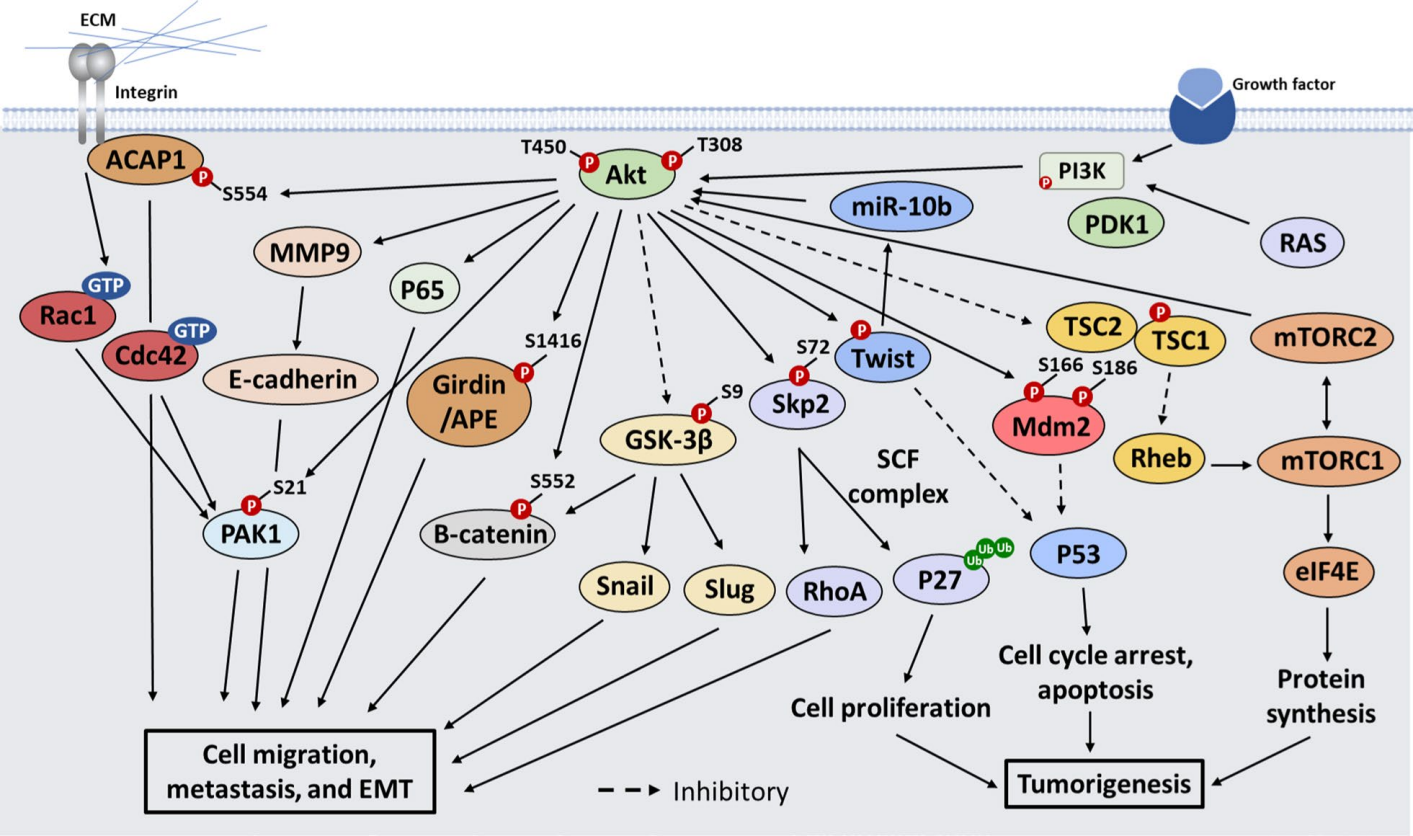
Akt regulates cell migration, EMT and metastasis through distinct mechanisms

Although more than 200 Akt substrates have been identified over the past decades, only a few have been validated to be critical in Akt-mediated cancer development. Akt regulates a plethora of biological processes, such as cell cycle regulation, cell survival, cellular senescence and epithelial-mesenchymal transition (EMT) for cancer formation, migration, invasion and metastasis by phosphorylating a variety of downstream effectors. When analyzing the characteristics of these downstream substrates, we found that these effectors commonly target cell cycle, apoptosis, EMT and cellular senescence, along with their regulation on other important hallmarks of cancer development (Fig. 2).

### Akt signaling in cell cycle regulation

Cell cycle is regulated by distinct protein machineries that are tightly controlled in the cells [49]. Alteration in cell cycle regulation is believed to be a key driving force for cancer development. Growth factors and cytokines provide important fuels needed for cell cycle progression and cell proliferation. The central player responsible for cell cycle control is Akt [3, 50]. Akt mainly regulates the transition from G1 phase to S phase, although it may also affect other phases of cell cycle. Extensive studies over the past decades have identified several Akt downstream effectors responsible for Akt-mediated cell cycle regulation and tumorigenesis.

1. **p21 and p27** Cyclin inhibitors, such as p21, p27 and p16, arrest cells in G1 phase by binding to these CDK complexes to inhibit their enzymatic activities. In order to control CDK kinase activity, p21 and p27 must stay in the nucleus to meet the cyclin/CDK complexes. Interestingly, p21 and p27 are phosphorylated by Akt at T145 and T157, respectively, and the phosphorylation of these proteins triggers their cytosolic localization, in turn inactivating their functions in the nucleus [51–53]. Phosphorylation of p21 is correlated with high Akt activity in advanced human breast cancer. Cytoplasmic p27 was found in primary human breast cancers in conjunction with Akt activation and was correlated with a poor patient prognosis [54, 55]. Moreover, the expression of p27 is downregulated in various human cancers, suggesting that p27 may be a tumor suppressor gene. This is supported by the genetic evidence that *p27* deficiency cooperates with *Pten* inactivation or *Smad3* loss to induce invasive prostate cancer and leukemia, respectively, although *p27* knockout mice do not develop overt tumor phenotypes [56, 57]. Thus, p27 loss in human is likely to play a tumor promoting effect.
2. **Foxo3a** Foxo3a, a member of the forkhead homeobox type O filmily of transcription factors, is an important downstream effector of the PI3K/AKT pathway. Foxo3a normally resides in the nucleus, but it delocalizes to the cytosol upon growth factor stimulation which mediated by Akt activation. Akt phosphorylates Foxo3a at T32, S253 and S315, resulting in cytosolic sequestration and inactivation of Foxo3a [58]. Inactivation or loss of Foxo3a is associated with many types of human cancers. Although the requirement of Foxo3a inactivation for Akt-driven tumorigenesis has not been addressed, Foxo3a inactivation seems to favor cancer development. Deficiency of *Foxo3a* alone in mice is not sufficient to initiate tumor development perhaps due to the compensatory mechanism from other Foxo family proteins, but simultaneous inactivation of *Foxo3a*, *Foxo1* and *Foxo4* in mice leads to the development of thymic lymphoma and hemangiomas [59]. In xenograft tumor model, *Foxo3a* silencing promotes breast cancer development, while its overexpression inhibits it [60]. Thus, Foxo3a is a tumor suppressor gene whose inactivation by Akt may lead to cancer initiation and progression.
3. **Skp2** Skp2, an F-box protein, forms a Skp2 SCF complex with Skp1, Cul-1 and Rbx1 [61, 62]. The Skp2 SCF complex displays E3 ubiquitin ligase activity, and the integrity of this complex is critical for its E3 ligase activity. Skp2 is a key determinant factor for this complex, as its expression levels are regulated by cell cycle. Skp2 binds to its substrates, such as p27, through its C-terminal LRR domain and triggers their ubiquitination and degradation [63, 64]. Although Skp2 serves as an upstream regulator for Akt K63-linked ubiquitination and activation, Skp2 stability and its activity can be regulated by Akt. Two earlier reports demonstrated that Akt is a bona fide kinase responsible for Skp2 phosphorylation [65, 66]. Akt interacts with and phosphorylates Skp2 at S72 located within the F-box domain. Phosphorylation of Skp2 at S72 by Akt prevents the interaction of Skp2 with Cdh1, thus abrogating Cdh1-mediated Skp2 ubiquitination and degradation [65]. In addition to affecting Skp2 stability, this phosphorylation also plays an important role in regulating Skp2 E3 ligase activity for cell cycle progression and Akt activation [11, 66].
  Similar to Akt, overexpression of Skp2 is detected in many types of human cancers. Interestingly, Skp2 overexpression is correlated with Akt hyperactivation [66–68], suggesting that Skp2 and Akt may act in a linear pathway or act in concert to facilitate cancer development. The notion is supported by two reports showing that transgenic mice expressing Skp2 in prostate also develop prostate intraepithelial neoplasia (PIN) similar to Akt1 transgenic mice, and that *Skp2* deficiency profoundly restricts prostate cancer and adrenal tumor formation upon *PTEN* inactivation [69, 70], suggesting that Skp2 is a critical player for PTEN/Akt-mediated tumorigenesis.
4. **Mdm2** Mdm2 is a major E3 ligase that triggers p53 ubiquitination and degradation [71, 72]. This notion is supported by genetic evidence showing that *Mdm2* deficiency causes embryonic lethality by upregulating p53 protein levels and that *p53* deficiency fully rescues this lethality [73]. Mdm2 normally resides in the nucleus to keep p53 protein levels low and inactive, but it is shuttled to the cytoplasm upon various stresses [71]. Notably, Mdm2 nuclear retention is regulated by Akt-mediated Mdm2 phosphorylation. Akt phosphorylates Mdm2 at S166 and S186, which is required for Mdm2 nuclear translocation [74, 75]. As a result, inhibiting Akt activity prevents Mdm2 nuclear translocation and triggers p53 ubiquitination and degradation, resulting in cell cycle arrest. Although the direct evidence for the involvement of Mdm2 in Akt-mediated cancer development is still lacking, Mdm2 S166D/S186D, but not wild-type Mdm2, synergizes with Neu overexpression to promote breast cancer development [76]. These results suggest that Akt-mediated Mdm2 phosphorylation plays an important role in promoting breast cancer development. However, it is unclear whether Mdm2 phosphorylation can directly regulate Mdm2 E3 ligase activity.
5. **GSK-3β** Glycogen synthase kinase-3β (GSK-3β) phosphorylates cyclin D1 at T286, which primes cyclin D for ubiquitination and degradation by the family of SCF E3 ligases, such as β-TrCP [77]. GSK-3β also inhibits cyclin D1 gene expression through indirectly by disrupting active β-catenin/TCF complex, which is known to bind to cyclin D1 promoter to induce its gene expression. GSK-3β is constitutively active in the cells but is inactivated by Akt. Akt phosphorylates GSK-3β at S9 to inactivate GSK-3β kinase activity [50]. Thus, Akt can promote cyclin D1 gene expression and protein stability through GSK-3β phosphorylation and inactivation to induce cell cycle progression.
6. **TSC1/TSC2 and mTOR** TSC1/TSC2 complex, in which TSC2 displays a GTPase activity, can antagonize mTOR activation by removing GTP from the GTP-bound Rheb. Intriguingly, the formation of TSC1/TSC2 complex is regulated by Akt. Akt phosphorylates TSC2 at multiple sites including S939, S1086/S1088 and T1422, a key event disrupting the TSC1/TSC2 complex and relieving its inhibition of Rheb and mTOR [55, 78, 79]. Collectively, the inactivation of TSC2 through Akt-mediated TSC2 phosphorylation leads to mTOR activation, in turn regulating cell growth and proliferation.
  mTOR regulates protein translation and cell cycle progression by regulating the phosphorylation of S6K and 4E-BP1. eIF4E activation appears to be a critical event for Akt-mediated cancer development, whereas the activation of S6K downstream ribosomal protein S6 (rpS6) is dispensable for it. In support of this notion, mutant 4E-BP1 overexpression rendering eIF4E inactivation attenuates lymphoma development in the transgenic mice expressing active *Akt* in immature T cells, but *rpS6* deficiency does not impact it [80]. Similarly, overexpression of mutant 4E-BP1 inhibits PI3KCA and K-Ras mutant tumor growth in the xenograft tumor model [81]. Thus, the mTOR-eIF4E pathway represents important therapeutic targets for cancer with aberrant Akt activation.
  Mice with one allele of *Tsc1* or *Tsc2* inactivation display mTOR hyperactivation and develop spontaneous liver cancers [82, 83]. Notably, *Tsc2* deficiency cooperates with one allele of *PTEN* inactivation to induce invasive prostate cancer [84]. In contrast, Rheb, a downstream target of the TSC1/TSC2 complex, is overexpressed in human cancers, and transgenic mice with Rheb overexpression in prostate exhibit mTOR hyperactivation and develop high grade PIN [85]. Thus, the Rheb/mTOR signaling plays an important role in cancer development.
7. **IKKα/NF-κB** The IKK complex consisting IKKα, IKKβ and IKKγ (also known as Nemo) regulates NF-κB activation and inflammatory response in response to various cytokines [86]. Interestingly, IKKα activity is also regulated by growth factors through Akt signaling activation. Phosphorylation IKKα at T23 by Akt is critical for IKKα kinase activation and subsequent NF-κB activation [87]. Although the in vivo role of IKKα T23 phosphorylation in cancer development has not been determined in mice, IKKα kinase activity is associated with mouse and human cancer development. Analyzing the human cancer sample specimen reveals that IKKα kinase activity is significantly correlated with cancer progression, and loss of IKKα kinase activity is shown to restrict prostate cancer progression and metastasis in *TRAMP* mice [88].

### Akt signaling in cellular senescence

In contrast to Akt-mediated oncogenic activities, Akt hyperactivation can also elicit cellular senescence to limit cell proliferation.

1. **p53/p21** Cellular senescence elicited by many stimuli is mostly associated with the induction of the p53/p21 pathway. Likewise, overexpression of active Akt1 and acute loss of *PTEN* triggers p53 and p21 induction, accompanied by cellular senescence. Moreover, inactivation of p53 or p21 expression can abolish this cellular senescence response, suggesting that Akt hyperactivation triggers p53/p21-dependent senescence [89]. In addition, Akt hyperactivation can also trigger p27-dependent cellular senescence, which strictly limits the ability of active Akt1 to induce invasive prostate cancer [90]. As such, *p27* deficiency abolishes this cellular senescence response and synergizes with Akt1 overexpression to induce invasive prostate cancer development [90].
2. **mTOR** Since Akt is known to antagonize p53 and p27 expression and activity as aforementioned, it is unclear why Akt hyperactivation upregulates p53 and p27 expression. Alimonti et al. provide a direct link between mTOR and p53 induction in cellular senescence upon acute *PTEN* inactivation [91]. However, p27 induction upon *PTEN* loss or Akt hyperactivation seems to occur independently of mTOR activation [90]. Acute *PTEN* inactivation leads to mTOR activation, which plays a critical role in Cap-dependent protein translation by regulating eIF4E and S6K activation, in turn enhancing p53 protein translation and cellular senescence [91]. Notably, overexpression of eIF4E also causes cellular senescence [92], suggesting that cellular senescence induced by mTOR hyperactivation likely through eIF4E activation.
3. **Skp2** Induction of cellular senescence appears to be an important barrier to restrict PTEN-Akt-mediated tumorigenesis upon *Skp2* deficiency. Interestingly, although *Skp2* deficiency on its own is not sufficient to trigger cellular senescence, it cooperates with *Pten* loss to trigger a novel p19Arf/p53-independent senescence to restrict cancer development [70]. Thus, Skp2 is also an important negative regulator for cellular senescence. In addition to being involved in Akt-mediated cancer development, Skp2 is also required for tumorigenesis upon *pRb* or *p19Arf* inactivation [70, 93], suggesting that Skp2 may serve as a common downstream effector for tumorigenesis driven by various oncogenic signals.
4. **Reactive Oxygen Species (ROS)** ROS has shown to play an important role in cellular senescence. Overexpression of active Akt in MEFs also triggers ROS production and cellular senescence, and Akt is shown to be required for Ras-mediated ROS production and cellular senescence [94]. In addition, ROS levels are regulated by oxygen consumption, MnSOD (manganese superoxide dismutase) and catalase. Gene expression of MnSOD and catalase is critically regulated by Foxo3a, and *Foxo3a* deficiency inhibits ROS production and cellular senescence upon *Ras* overexpression. Interestingly, overexpression of active Akt enhances oxygen consumption and reduces MnSOD and catalase expression, which are correlated with Foxo3a inactivation, ROS production and cellular senescence [94]. Accordingly, Ras/Akt triggers ROS production and cellular senescence likely through negatively regulating Foxo3a activation.

### Akt in cancer metastasis

Cell migration and invasion are critical steps for cancer metastasis, which accounts for the major cause of the death in cancer patients. Although Akt is known to be a critical player in cell migration and metastasis [50], its role in these processes appears to be controversial. The distinct Akt isoforms and various cell types used in the study may explain this discrepancy. Akt1 appears to promote cell migration in fibroblast cells, whereas Akt2 inhibits it. However, Akt1 inhibits breast cancer migration and metastasis, while Akt2 exhibits an opposite phenomena [50]. However, the systematic versus cell-autonomous deletion of Akt isoforms could display distinct impacts on cancer progression as aforementioned.

Nevertheless, these lines of evidence underscore the important role of Akt in cell migration and metastasis.

1. **Rho GTPases** The control of cell migration and metastasis by Akt can act through several distinct mechanisms. Rho GTPases including RhoA, Rac1 and Cdc42 are major players in cell migration and metastasis by regulating actin polymerization and cytoskeleton reorganization [95]. Akt is shown to regulate the activity of PAK1, a downstream effector of Rac1 and Cdc42 involved in cell migration, by phosphorylating PAK1 at S21, although the functional role of this phosphorylation in cell migration remains to be determined [96]. Overexpression of RhoA gene and protein is found in various metastatic human cancers. Interestingly, RhoA gene expression is induced by the Skp2/Myc/Miz1 complex, whose overexpression is correlated with RhoA expression in metastatic cancer [67]. Overexpression of Skp2 promotes cell migration and metastasis, while its deficiency inhibits these processes [67]. Notably, restoration of RhoA expression in *Skp2* deficiency cancer cells fully rescues the defects in cancer cell migration and invasion [67]. Thus, RhoA is a relevant downstream effector for Skp2-mediated cell migration and metastasis. Phosphorylation of Skp2 by Akt at S72 is critical for Skp2-mediated cell migration, as Skp2 S72A mutant loses its ability to promote cell migration [66]. Thus, Akt may regulate cell migration partly through regulating Skp2 phosphorylation.
2. **Girdin/APE and ACAP1** Akt may also positively regulate cell migration by phosphorylating Girdin/APE and ACAP1. Girdin/APE plays a crucial role in the formation of stress fiber and lamellipodia, while ACAP1 functions as a transporter effector involved in integrin β1 recycling and cell migration [97, 98]. Phosphorylation of Girdin by Akt at S1416 accumulates in leading edge of migrating cells and is critical for cell migration and lamellipodia formation [97]. Similarly, phosphorylation of ACAP1 by Akt at S554 is required for the interaction of ACAP1 with integrin β1 and in turn regulates cell migration [98].

In contrast, Akt1 is shown to reduce cell migration and invasion in breast cancer cells. The inhibitory effect of Akt1 on cell migration depends on its ability to phosphorylate Mdm2 at S166 and S186, which in turn triggers ubiquitination and degradation of NFAT transcription factor [99]. Akt1 can also inhibit breast cancer cell migration and invasion by phosphorylating paladin at S507, an actin binding protein involved in actin cytoskeleton organization and cell migration [100]. Another potential mechanism by which Akt1 inhibits breast cancer migration may act through the inhibitory role of Akt1 on ERK activation [101]. Unlike Akt1, Akt2 does not induce paladin phosphorylation and fails to inhibit breast cancer cell migration, instead it promotes breast cancer cell migration [100, 101]. Future study is warranted to understand how Akt2 regulates cell migration.

### Akt signaling in EMT

Upon its activation, Akt phosphorylates number of substrates, thereby affecting a series of cellular and physiological processes, these changes may induce the EMT process.

1. **Twist** Twist is a basic helix-loop-helix often acts as a transcription factor with a strong ability to induce the EMT by amplifying the expression of N-cadherin, Bmi1, Akt2 and Y-box binding protein-1 (YB-1). Vichalkovski et al*.* found that activated Akt upregulated the expression of phosphorylated Twist and subsequently alleviated the induction of p53 caused by chemotherapy-induced DNA damage, resulting in the inhibition of apoptosis [102]. In oral squamous cell carcinoma, there is a positive correlation between the expression of Twist and phosphorylated Akt [103]. Notably, Twist can also modulate Akt signaling activation. Liu et al*.* reported that Twist induced miR-10b expression and subsequently promoted the phosphorylation of Akt, thereby increasing the invasiveness of gastric cancer cells [104]. Another study reported that Twist can enhance the biological activity of Akt primarily through its interaction with the E-box element in the Akt2 promoter [105]. Thus, Akt and Twist are involved in a positive feedback loop, resulting in a series of events that enhance their pro-EMT function.
2. **Snail and Slug** Snail and Slug which shuttle between cytoplasm and nucleus are DNA binding proteins with zinc finger structures to locate an E-box that serves as the promoter upstream of E-cadherin; then bind to the E-box elements to trigger EMT by inhibiting E-cadherin gene [106]. Activation of PI3K/Akt signaling can inhibit the degradation of Snail by phosphorylating and inactivating GSK-3β or enhance Snail expression through activation of NF-κB [107]. The excessive activation of Akt results in S9 phosphorylation and inactivation of GSK-3β, leading to preventing the degradation of Snail [108]. Like Snail, Slug is also an important modulator of EMT in cancer cells [109]. GSK-3β inactivation after PI3K/Akt activation leads to Slug upregulation, followed by the induction of the EMT [110].
3. **ECM and MMPs** Matrix-degrading proteases disrupt the basement membrane and interstitial matrix thereby allowing for cancer cells to invade, promote the release and activation of cytokines that bind to the extracellular matrix and split the extracellular domain of E-cadherin [111, 112]. Upon activation of PDK1 and PI3K, Akt is activated and increases the expression of p65, a subunit NF-κB, thus leading to the downregulation of E-cadherin expression [113]. In head and neck squamous cell carcinoma and gastric cancer, the activation and inhibition of PI3K/Akt alters the expression of MMP9 to regulate E-cadherin, thus modulating cell invasion and migration [114, 115].
4. **Wnt/β-catenin signaling pathway** Upon Wnt signaling engagement, β-catenin combines with intracellular domain of E-cadherin to form a complex. The PI3K/Akt pathway positively regulates Wnt/β-catenin in two distinct manners, both of which contribute to the induction of EMT. The first mechanism involves the movement of β-catenin into the nucleus, typically accompanied by Akt phosphorylation. Activated Akt then phosphorylates S552 in β-catenin leading to an increase in its transcriptional activity [116]. In addition, GSK-3β inactivation by Akt elevates the stability of both β-catenin and Snail. Altogether, the activation of Akt then increases intracellular β-catenin levels.

### Akt signaling in epigenetic regulation

The epigenetic modifiers including a series of proteins and enzymes, which influence chromatin reading, writing and erasure, have been shown to participate in oncogenicity of PI3K/Akt signaling in cancer. Akt has been shown to induce the S21 phosphorylation of Enhancer Of Zeste 2 Polycomb Repressive Complex 2 Subunit (EZH2), which blocks EZH2 methyltransferase activity towards histone H3 K27 trimethylation [117]. Akt also phosphorylates histone methyltransferase WHSC1 and stabilizes it for inducing RICTOR gene expression and promoting prostate cancer metastasis [118]. Akt can phosphorylate DNA methyltransferase DNMT1 at S143 resulting in DNMT1 stabilization [119], although it remains to be determined whether this phosphorylation directly impact on DNA methyltransfease activity of DNMT1. Furthermore, activation of Akt has been shown to induce histone lysine acetyltransferase activity of CBP and p300 by directly phosphorylating CBP and p300 at S1834 and T1871, respectively [120, 121]. It will be of significance to further uncover the complexity of crosstalk between Akt and epigenetic modifying enzymes in transcriptional regulation and tumorigenesis.

## Therapeutic implications for cancer intervention by targeting Akt and its upstream regulators and downstream effectors

**Fig. 3:**
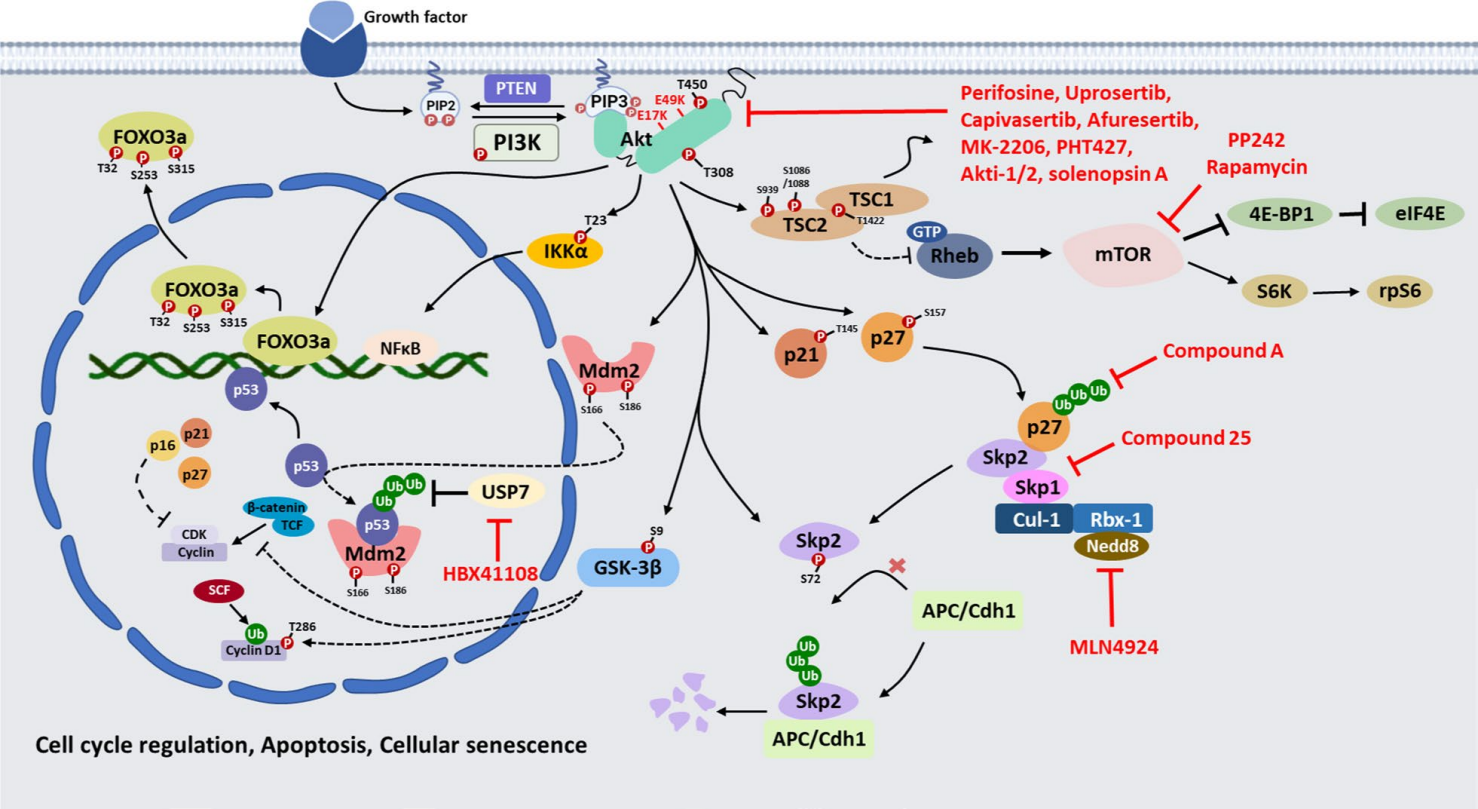
Downstream effectors of Akt and the potential therapeutic inhibitors

The finding that Akt and its upstream regulators and downstream effectors play pivotal roles in diverse oncogenic processes suggests that targeting Akt and its regulations may offer potential opportunities for cancer therapeutic intervention, exemplified by numerous Akt inhibitors shown to be effective in preclinical models and currently tested in clinical trials (Fig. 3 and Table 1).

**Table 1:** Examples of small-molecular inhibitor targeting Akt and its downstream effectors for human cancer therapy

Name	Target	Effect	Tumor	Clinical trail
Uprosertib (GSK2141795)	Akt1/2/3	ATP-competitive pan-Akt inhibitor	Endometrial carcinoma; myeloma; melanoma; hematopoietic and lymphoid cell neoplasm; malignant solid neoplasm	Phase I/II
Capivasertib (AZD5363)	Akt1/2/3;P70S6K/PKA	ATP-competitive pan-Akt inhibitor	B-cell non-hodgkin lymphoma; breast; prostate; solid and hematological tumors	Phase I/II/III
Ipatasertib (GDC-0068)	Akt1/2/3	ATP-competitive pan-Akt inhibitor	Breast; head and neck carcinoma; solid tumors; gastric; prostate; ovarian; NSCLC	Phase I/II/III
Afuresertib (GSK2110183)	Akt1/2/3	ATP-competitive pan-Akt inhibitor	Breast; prostate; ovarian; solid tumors	Phase I/II
GSK690693	Akt1/2/3;PKA, PrkX, PKC	ATP-competitive pan-Akt inhibitor	Hematologic malignancies; solid tumors; lymphoma	Phase I
LY2780301	Akt1/2/3;P70S6K	ATP-competitive pan-Akt inhibitor	Breast; solid tumors; non-Hodgkin’s lymphoma; metastatic cancers	Phase I/II
Perifosine (KRX-0401)	Akt1/2	Allosteric Akt inhibitor	Brain; prostate; pancreatic; melanoma; renal cell carcinoma; breast; NSCLC; myeloma; solid tumors	Phase I/II
MK-2206	Akt1/2/3	Allosteric Akt inhibitor	Solid tumors; lymphomas; breast; colorectal; gall bladder; melanoma; nsclc; oral; ovarian; pancreatic; prostate	Phase I/II
Miransertib (ARQ 092)	Akt1/2/3	Allosteric Akt inhibitor	Proteus syndrome; solid tumors; lymphomas; ovarian; endometrial	Phase I/II
ARQ 751	Akt1/2/3	Allosteric Akt inhibitor	Solid tumors	Phase I
TAS117	Akt1/2/3	Allosteric Akt inhibitor	Solid tumors	Phase II
BAY1125976	Akt1/2	Allosteric Akt inhibitor	Neoplasms	Phase I
PHT427	Akt1/2/3	Allosteric Akt inhibitor	NA	NA
Akti-1/2	Akt1/2	Allosteric Akt inhibitor	NA	NA
Solenopsin	Akt	An alkaloid component of fire ant venom	NA	NA
Rapamycin; rapaloges	mTORC1	Allosteric mTORC1 inhibitor	Bladder; pancreatic; solid tumors; lymphoma; leukemia; prostate; brain; NSCLC	Phase I/II
PP242	mTORC1/C2	Inhibiting mTOR and eIF4E activation	NA	NA
Compound A	Skp2	Inhibiting Skp2 SCF E3 ligase activity to prevent p27 ubiquitination and degradation	NA	NA
Compound 25	Skp2	Preventing the assembly of newly synthesized Skp2-Skp1 complex by binding to Skp2 to inactivate its E3 ligase activity	NA	NA
Pevonedistat (MLN4924)	Nedd8-activating enzyme	Inhibiting neddylation of Cul-1 and the formation Skp2 SCF complex	Leukemia; myeloma; melanoma; solid tumors	Phase I/II
Nutlin-3	Mdm2	Occupation of the binding site of p53 in MDM2	NA	NA
HBX41108	USP7	Targeting USP7 to prevent MDM2 deubiquitination and promote MDM2 degradation	NA	NA

NA: not applicable

### Targeting on Akt

Several ATP-competitive and allosteric Akt inhibitors have been synthetized and most of them showed a satisfactory safety profile in clinical trials. ATP-competitive inhibitors, such as Uprosertib/GSK21417, Capivasertib/AZD53, Ipatasertib/GDC-0068, Afuresertib/GSK2110183 and GSK690693; LY2780301 competes with ATP to occupy Akt kinase ATP binding site, preventing the phosphorylation activation of Akt. **Uprosertib (GSK2141795)** promotes cisplatin-induced apoptosis and ablates phosphorylation of proline-rich Akt substrates in an ovarian cancer xenograft mouse model [122, 123]. **Capivasertib (AZD5363)** shows a constructive pharmacokinetic and toxicity profile in a BT474c breast cancer xenograft mouse model [124] and exhibits a promising efficacy in targeting castration resistant prostate cancer[125], Her2^+^ breast cancer models and tumors harboring the Akt1 E17K mutation [126]. **Ipatasertib (GDC-0068)** is also effective both in cancer cell lines and in xenograft models with activation of Akt due to complete loss of PTEN (PTEN-null), decreased expression of PTEN or mutations in PIK3CA [127]. **Afuresertib (GSK2110183)** is widely studied in clinical trials in different types of cancer including ovarian cancer, prostate cancer, triple-negative breast cancer and HR^+^/HER2^−^ breast cancer [128]. **GSK690693** blocks Akt signaling and induces apoptosis in various hematologic malignancies, especially ALL and some lymphomas [129]. **LY2780301** reduces growth of a wide range of cancer cells and tumor xenografts and exhibits synergism with various targeted therapies [130]. Allosteric inhibitors (Perifosine/KRX-0401; MK-2206; Miransertib/ARQ092; ARQ 751; TAS-117; BAY1125976; PHT427; Akti-1/2) by blocking the Akt translocation to the plasma membrane are employed in advanced solid tumors and tested in many early phase trials. **Perifosine (KRX-0401)** exerts a marked cytotoxic effect against multiple human tumor cell lines and preclinical cancer models [131–133]. Another study revealed that small molecule inhibitors targeting PI3K/Akt have been identified and tested in clinical trials [134]. **MK2206** has been developed and proven to be effective in small cell lung cancer [135, 136]. **Miransertib (ARQ092)** reduces the phosphorylation of Akt downstream substrates glycogen synthase kinase 3α (GSK-3α) and Akt activation in cancers conferring Akt1-E17K [137, 138]. Notably, administration of Miranbserib reduces PIK3CA-related overgrowth spectrum disorder in vivo studies and clinical studies [139]. **ARQ 751** holds great potential in treating patients with solid tumors harboring mutations in the PTEN/PI3K/Akt pathway [138]. **TAS-117** inhibits the proliferation of various human cancer cell lines in vitro, including breast, endometrial, lung and ovarian cancer cells with Akt2 or human epidermal growth factor type 2 (HER2) gene amplification, PIK3C mutations or PTEN loss [140]. **BAY1125976** inhibits broadly growth of human cancer cell lines and tumor xenografts, including the KPL-4 breast cancer model (PIK3CA H1074R mutant), the MCF7 and HBCx-2 breast cancer models, the Akt E17 K mutation-driven prostate cancer (LAPC-4) and anal cancer (AXF984) model [141]. **PHT-427** inhibited Akt and PDKP1 signaling and their downstream targets and the growth of human tumor xenografts [142]. **Akti-1/2** blocks the PH domain and activation of Akt1 and Akt2 but not Akt3 [143, 144] and its efficiency has been validated in hepatocytes [145], breast tumor cells [146] and chronic lymphocytic leukemia cells [147, 148]. Finally, **solenopsin A,** an alkaloid component of fire ant venom, has been reported to be an Akt inhibitor that can antagonize Akt cellular activity in vitro [149]. Small molecule analogs of solenopsin has been demonstrated for the treatment of various cancers including melanoma and angiosarcoma [150].

Proteolysis targeting chimeras (PROTACs) are promising new therapeutic modalities, which degrade the target protein through the corresponding endogenous ubiquitin proteasome system (UPS). A few PROTAC Akt degraders have been reported in recent years, which may offer more effective therapeutic strategy than pharmacological inhibition of Akt kinase activity. INY-03-041 is the first Akt PROTAC using GDC-0068 as an Akt binding moiety conjugated to lenalidomide (Cereblon ligand), a recruiter of the E3 ubiquitin ligase substrate adaptor Cereblon (CRBN) and degrade all three isoforms [151]. Of note, INY-03-041 displays more potent effect on suppressing cancer cell proliferation than GDC-0068. Other Akt degraders, MS21 and MS143, which are von Hippel–Lindau (VHL)-recruiting PROTACs based on the Akt inhibitor AZD5363, induce rapid and robust Akt degradation leading to suppressing cancer cell growth and tumor growth in vivo in a xenograft model without causing apparent toxicity [152]. Further optimization of these degraders will be needed to provide a potential Akt degradation therapy for targeting cancer and various other diseases associate with Akt activation

### Targeting on mTOR

The finding that mTOR is hyperactive in human cancers and plays a key role in Akt-mediated cancer development in mouse models suggests that mTOR is a potential target for human cancers. **Rapamycin** and its derivatives are shown to be effective in certain types of cancer in preclinical mouse model. However, they are not effective in most cancers due to the mechanisms associated with their partial inhibition on mTORC1 and mTORC2 activation that plays a prominent role in driving resistance to Rapamycin in tumors through continued phosphorylation of Akt on Ser 473. Other related rapalogues, such as Everolimus, Temsirolimus, and Ridaforolimus, targeting PI3K/AKT/mTORC1 axis have been tested in clinical trials, and successful clinical trials have resulted in FDA approval for Everolimus and Temsirolimus in the treatment of renal cell carcinoma and selected breast cancers. **PP242**, an active site inhibitor of mTOR, profoundly inhibits Akt-mediated lymphomagenesis by inhibiting mTOR and eIF4E activation [80]. Since eIF4E is also required for Akt-mediated cancers, small molecule inhibitors targeting eIF4E can be also applied to cancer with deregulated Akt activity. Toward this direction, a small molecule targeting eIF4E has been developed and shown to be effective in cell based assay [153]. The second generation of small molecule inhibitors including AZD-8055, MLN0128 (INK128), PP30 and XL-388 targeting both mTORC1 and mTORC2 have been developed and tested in preclinical mouse model with promising efficacy and clinical trials [154–157]. Since PI3K and mTOR, which share highly similar catalytic domains, dual inhibitors such as BEZ235, GDC-0980, XL765 (SAR245409), GSK2126458 and PF-05212384 (PKI-578) offering a more complete inhibition of the pathway, have been developed to tackle resistance to mTOR kinase inhibitors and rapalogues [158].

### Targeting on other Akt upstream regulators and downstream effectors

We suggest that targeting Skp2, an upstream regulator for Akt, can be another potential approach for human cancer treatment on the basis of the findings from our group and others that Skp2 is required for cancer development in diverse genetic tumor models [33, 70, 159–161]. Consistent with this notion, several small molecules targeting the Skp2 SCF complex were recently identified and proven to be promising for treating human cancers. One study shows that **compound A**, a small molecule targeting Skp2 SCF E3 ligase activity towards p27 ubiquitination, causes cell arrest, apoptosis and autophagy in leukemia cells [162]. By using structural-based high-throughput virtual screening technologies, our group recently identified **compound 25** to be another small-molecule that targets Skp2 SCF complex for inactivation. It specifically binds to Skp2 and prevents its binding with Skp1, thereby disrupting the Skp2 SCF complex and its E3 ligase activity toward Akt and p27. Not only compound 25 is a potent inhibitor against cancer cell growth while sparing the normal ones, it can also restrict cancer stem cell population and self-renewal ability leading to suppressing cancer progression in mouse models [163]. Other studies demonstrated that **Pevonedistat** (**MLN4924)**, a small molecule inhibitor targeting Nedd8-activating enzyme to disrupt neddylation of Cul-1 and the formation Skp2 SCF complex [164], remarkably induces regression of various tumors in preclinical mouse model by inducing apoptosis or senescence [70, 164]. These studies therefore call for a need to develop better Skp2 small molecule inhibitors for preclinical and clinical studies. Apart from targeting Skp2, development of specific inhibitors targeting Akt upstream regulators, such as TRAF6, SETDB1 and JMJD2A, may also serve as a novel and innovative strategy for eradicating hyperactive Akt-driven human cancers.

Among Akt downstream effectors, Mdm2, IKKβ, PAK1 and Rho GTPases may represent potential targets for human cancers. In particular, Mdm2 is a highly relevant target for human cancers, as Mdm2 is overexpressed in numerous human cancers and its overexpression facilitates tumor formation in transgenic mouse models. Indeed, various MDM2 small molecule inhibitors including **Nutlin-3** have been developed and exhibited a promising effect in numerous preclinical models [165–168]. The clinical trials assessing their efficacy have been initiated [169–171]. Since USP7 is a deubiquitinating enzyme that interacts with Mdm2 and prevents its ubiquitination and degradation [172, 173], targeting USP7 can be an alternative approach for cancer treatment. Indeed, in vitro assay reveals that **HBX41108**, a USP7 inhibitor, can induce p53-dependent apoptosis of HCT116 cancer cells [174]. Future experiments will be required to test the efficacy of this compound in preventing tumor growth in vivo.

Overall, multiple strategies targeting of the entire Akt signaling pathway alone or in combination with other standard of care therapies may offer promising solutions for future cancer targeting. Hence, Akt, as a potential therapeutic target of cancer, should continue to draw great attention for understanding its regulation and the development of a variety of Akt and its pathway inhibitors for cancer prevention and treatment.

## Conclusions

The intensive research efforts on studying Akt signaling during last two decades have significantly advanced our current understandings of how oncogenic Akt is activated and how it transmits the signal to downstream effectors to participate in various biological processes. Notably, hundreds of Akt substrates have been identified mostly through a bioinformatic approach, which predicts potential Akt substrates by analyzing the Akt consensus phosphorylation motif. However, only a few of them have been genetically proven to be relevant substrates responsible for Akt actions. Most of its substrates remains to be determined what their functional relevance in Akt signaling is. Addressing these questions will lead to identifying important drug targets for various human diseases associated with deregulated Akt signaling activation.

Several protein candidates are proven to be critical for Akt-mediated cancer development in preclinical mouse models as previously mentioned. Future research efforts towards developing small molecule inhibitors targeting these proteins will be warranted and important to further validate this notion presented above. Moreover, developing small molecule inhibitors specifically target different Akt isoforms will provide better therapeutic efficacy on cancer treatment while reducing side effect, given the distinct effects of Akt isoforms on cancer metastasis and the essential role of Akt in normal cell functions.

Finally, the detailed mechanism by which the distinct roles of three Akt isoforms play in cell migration and metastasis from various tissue types and how systematic versus cell-autonomous deletions of Akt isoforms distinctly impact cancer features should be also addressed. We believe that identifying the substrate specificity of Akt isoforms will provide novel insights into how Akt isoforms play distinct roles in a variety of biological processes.

## Data Availability

Not applicable.

## Abbreviations

PI3K: Phosphatidylinositol 3-kinase
PIP3: Phosphoinositol 3, 4, 5-triphosphate
PIP2: Phosphoinositol 4, 5-biphosphate
PKB: Protein kinase B
PH: Pleckstrin homology
PTMs: Posttranslational modifications
mTORC2: MTOR complex 2
mTOR: Mammalian target of rapamycin
mSIN1: Mammalian stress-activated protein kinase interacting protein 1
RICTOR: Rapamycin-insensitive companion of mTOR
mLST8: Mammalian lethal with sec-13 protein 8
ER: Endoplasmic reticulum
CDK: Cyclin dependent kinase
PP2A: Protein phosphatase 2A
PHLPP: PH domain leucine-rich repeat protein phosphatases
HIF: Hypoxia-inducible factor
EMT: Epithelial-mesenchymal transition
NSCLC: Non-small cell lung cancer
TTC3: Tetratricopeptide repeat domain 3
BRCA1: Breast cancer susceptibility gene 1
CHIP: Chaperone-associated ubiquitin ligase
EZH2: Zeste 2 Polycomb Repressive Complex 2 Subunit
EMT: Epithelial-mesenchymal transition
PIN: Prostate intraepithelial neoplasia
GSK-3α: Glycogen synthase kinase-3α
GSK-3β: Glycogen synthase kinase-3β
rpS6: Ribosomal protein S6
ROS: Reactive oxygen species
MnSOD: Manganese superoxide dismutase
YB-1: Y-box binding protein-1
HER2: Human epidermal growth factor type-2

## References

[1] Cantley LC (2002). The phosphoinositide 3-kinase pathway. Science.

[2] Hennessy BT (2005). Exploiting the PI3K/AKT pathway for cancer drug discovery. Nat Rev Drug Discov.

[3] Manning BD, Cantley LC (2007). AKT/PKB signaling: navigating downstream. Cell.

[4] Yang WL (2010). Regulation of Akt signaling activation by ubiquitination. Cell Cycle.

[5] Dummler B, Hemmings BA (2007). Physiological roles of PKB/Akt isoforms in development and disease. Biochem Soc Trans.

[6] Gonzalez E, McGraw TE (2009). The Akt kinases: isoform specificity in metabolism and cancer. Cell Cycle.

[7] Oeck S (2017). Activating Akt1 mutations alter DNA double strand break repair and radiosensitivity. Sci Rep.

[8] Dillon RL (2009). Akt1 and akt2 play distinct roles in the initiation and metastatic phases of mammary tumor progression. Cancer Res.

[9] Hutchinson JN (2004). Activation of Akt-1 (PKB-alpha) can accelerate ErbB-2-mediated mammary tumorigenesis but suppresses tumor invasion. Cancer Res.

[10] Chen X (2020). Cell-autonomous versus systemic Akt isoform deletions uncovered new roles for Akt1 and Akt2 in breast cancer. Mol Cell.

[11] Chan CH (2012). The Skp2-SCF E3 ligase regulates Akt ubiquitination, glycolysis, herceptin sensitivity, and tumorigenesis. Cell.

[12] Guo J (2016). pVHL suppresses kinase activity of Akt in a proline-hydroxylation-dependent manner. Science.

[13] Yang WL (2009). The E3 ligase TRAF6 regulates Akt ubiquitination and activation. Science.

[14] Sundaresan NR (2011). The deacetylase SIRT1 promotes membrane localization and activation of Akt and PDK1 during tumorigenesis and cardiac hypertrophy. Sci Signal.

[15] Boulbes DR, Shaiken T, dos Sarbassov D (2011). Endoplasmic reticulum is a main localization site of mTORC2. Biochem Biophys Res Commun.

[16] Sarbassov DD, Ali SM, Sabatini DM (2005). Growing roles for the mTOR pathway. Curr Opin Cell Biol.

[17] Guertin DA, Sabatini DM (2007). Defining the role of mTOR in cancer. Cancer Cell.

[18] Facchinetti V (2008). The mammalian target of rapamycin complex 2 controls folding and stability of Akt and protein kinase C. EMBO J.

[19] Liu P (2014). Cell-cycle-regulated activation of Akt kinase by phosphorylation at its carboxyl terminus. Nature.

[20] Di Cristofano A, Pandolfi PP (2000). The multiple roles of PTEN in tumor suppression. Cell.

[21] Salmena L, Carracedo A, Pandolfi PP (2008). Tenets of PTEN tumor suppression. Cell.

[22] Alessi DR (1996). Mechanism of activation of protein kinase B by insulin and IGF-1. EMBO J.

[23] Kuo YC (2008). Regulation of phosphorylation of Thr-308 of Akt, cell proliferation, and survival by the B55alpha regulatory subunit targeting of the protein phosphatase 2A holoenzyme to Akt. J Biol Chem.

[24] Padmanabhan S (2009). A PP2A regulatory subunit regulates *C. elegans* insulin/IGF-1 signaling by modulating AKT-1 phosphorylation. Cell.

[25] Gao T, Furnari F, Newton AC (2005). PHLPP: a phosphatase that directly dephosphorylates Akt, promotes apoptosis, and suppresses tumor growth. Mol Cell.

[26] Brognard J (2007). PHLPP and a second isoform, PHLPP2, differentially attenuate the amplitude of Akt signaling by regulating distinct Akt isoforms. Mol Cell.

[27] Chen M (2011). Identification of PHLPP1 as a tumor suppressor reveals the role of feedback activation in PTEN-mutant prostate cancer progression. Cancer Cell.

[28] Strocchi S (2022). The multifaceted role of EGLN family prolyl hydroxylases in cancer: going beyond HIF regulation. Oncogene.

[29] Voulgarelis M, Tsichlis PN (2016). Proline hydroxylation linked to Akt activation. Science.

[30] Huang BX, Kim HY (2006). Interdomain conformational changes in Akt activation revealed by chemical cross-linking and tandem mass spectrometry. Mol Cell Proteomics.

[31] Yang WL, Zhang X, Lin HK (2010). Emerging role of Lys-63 ubiquitination in protein kinase and phosphatase activation and cancer development. Oncogene.

[32] Fan CD (2013). Ubiquitin-dependent regulation of phospho-AKT dynamics by the ubiquitin E3 ligase, NEDD4-1, in the insulin-like growth factor-1 response. J Biol Chem.

[33] Chan CH (2012). The Skp2-SCF E3 ligase regulates Akt ubiquitination, glycolysis, herceptin sensitivity, and tumorigenesis. Cell.

[34] Li W (2013). TRAF4 is a critical molecule for Akt activation in lung cancer. Cancer Res.

[35] Yang WL (2013). Cycles of ubiquitination and deubiquitination critically regulate growth factor-mediated activation of Akt signaling. Science signaling.

[36] Lim JH (2012). CYLD negatively regulates transforming growth factor-beta-signalling via deubiquitinating Akt. Nat Commun.

[37] Wang Q (2019). Regulation of the expression of DAPK1 by SUMO pathway. Biomolecules.

[38] Guo J (2019). AKT methylation by SETDB1 promotes AKT kinase activity and oncogenic functions. Nat Cell Biol.

[39] Noguchi M, Hirata N, Suizu F (2014). The links between AKT and two intracellular proteolytic cascades: ubiquitination and autophagy. Biochim Biophys Acta.

[40] Xiang T (2008). Negative regulation of AKT activation by BRCA1. Cancer Res.

[41] Bae S (2012). Akt is negatively regulated by the MULAN E3 ligase. Cell Res.

[42] Dickey CA (2008). Akt and CHIP coregulate tau degradation through coordinated interactions. Proc Natl Acad Sci U S A.

[43] Gareau JR, Lima CD (2010). The SUMO pathway: emerging mechanisms that shape specificity, conjugation and recognition. Nat Rev Mol Cell Biol.

[44] Li R (2013). Akt SUMOylation regulates cell proliferation and tumorigenesis. Cancer Res.

[45] Risso G (2013). Modification of Akt by SUMO conjugation regulates alternative splicing and cell cycle. Cell Cycle.

[46] Lin CH, Liu SY, Lee EH (2016). SUMO modification of Akt regulates global SUMOylation and substrate SUMOylation specificity through Akt phosphorylation of Ubc9 and SUMO1. Oncogene.

[47] Wang S (2012). Extensive crosstalk between O-GlcNAcylation and phosphorylation regulates Akt signaling. PLoS ONE.

[48] Heath JM (2014). Activation of AKT by O-linked N-acetylglucosamine induces vascular calcification in diabetes mellitus. Circ Res.

[49] Davidson G, Niehrs C (2010). Emerging links between CDK cell cycle regulators and Wnt signaling. Trends Cell Biol.

[50] Qiao M, Sheng S, Pardee AB (2008). Metastasis and AKT activation. Cell Cycle.

[51] Zhou BP (2001). Cytoplasmic localization of p21Cip1/WAF1 by Akt-induced phosphorylation in HER-2/neu-overexpressing cells. Nat Cell Biol.

[52] Reed SI (2002). Keeping p27(Kip1) in the cytoplasm: a second front in cancer's war on p27. Cell Cycle.

[53] Zhou BP, Hung MC (2002). Novel targets of Akt, p21(Cipl/WAF1), and MDM2. Semin Oncol.

[54] Liang J (2002). PKB/Akt phosphorylates p27, impairs nuclear import of p27 and opposes p27-mediated G1 arrest. Nat Med.

[55] Inoki K (2002). TSC2 is phosphorylated and inhibited by Akt and suppresses mTOR signalling. Nat Cell Biol.

[56] Di Cristofano A (2001). Pten and p27KIP1 cooperate in prostate cancer tumor suppression in the mouse. Nat Genet.

[57] Wolfraim LA (2004). Loss of Smad3 in acute T-cell lymphoblastic leukemia. N Engl J Med.

[58] Brunet A (1999). Akt promotes cell survival by phosphorylating and inhibiting a Forkhead transcription factor. Cell.

[59] Paik JH (2007). FoxOs are lineage-restricted redundant tumor suppressors and regulate endothelial cell homeostasis. Cell.

[60] Hu MC (2004). IkappaB kinase promotes tumorigenesis through inhibition of forkhead FOXO3a. Cell.

[61] Nakayama KI, Nakayama K (2005). Regulation of the cell cycle by SCF-type ubiquitin ligases. Semin Cell Dev Biol.

[62] Nakayama KI, Nakayama K (2006). Ubiquitin ligases: cell-cycle control and cancer. Nat Rev Cancer.

[63] Nakayama K (2000). Targeted disruption of Skp2 results in accumulation of cyclin E and p27(Kip1), polyploidy and centrosome overduplication. Embo J.

[64] Nakayama K (2004). Skp2-mediated degradation of p27 regulates progression into mitosis. Dev Cell.

[65] Gao D (2009). Phosphorylation by Akt1 promotes cytoplasmic localization of Skp2 and impairs APCCdh1-mediated Skp2 destruction. Nat Cell Biol.

[66] Lin HK (2009). Phosphorylation-dependent regulation of cytosolic localization and oncogenic function of Skp2 by Akt/PKB. Nat Cell Biol.

[67] Chan CH (2010). Deciphering the transcriptional complex critical for RhoA gene expression and cancer metastasis. Nat Cell Biol.

[68] Chan CH (2010). Regulation of Skp2 expression and activity and its role in cancer progression. ScientificWorldJournal.

[69] Shim EH (2003). Expression of the F-box protein SKP2 induces hyperplasia, dysplasia, and low-grade carcinoma in the mouse prostate. Cancer Res.

[70] Lin HK (2010). Skp2 targeting suppresses tumorigenesis by Arf-p53-independent cellular senescence. Nature.

[71] Kruse JP, Gu W (2009). Modes of p53 regulation. Cell.

[72] Manfredi JJ (2010). The Mdm2-p53 relationship evolves: Mdm2 swings both ways as an oncogene and a tumor suppressor. Genes Dev.

[73] Montes de Oca Luna R, Wagner DS, Lozano G (1995). Rescue of early embryonic lethality in mdm2-deficient mice by deletion of p53. Nature.

[74] Zhou BP (2001). HER-2/neu induces p53 ubiquitination via Akt-mediated MDM2 phosphorylation. Nat Cell Biol.

[75] Ashcroft M (2002). Phosphorylation of HDM2 by Akt. Oncogene.

[76] Cheng X (2010). Activation of murine double minute 2 by Akt in mammary epithelium delays mammary involution and accelerates mammary tumorigenesis. Cancer Res.

[77] Takahashi-Yanaga F, Sasaguri T (2008). GSK-3beta regulates cyclin D1 expression: a new target for chemotherapy. Cell Signal.

[78] Manning BD (2002). Identification of the tuberous sclerosis complex-2 tumor suppressor gene product tuberin as a target of the phosphoinositide 3-kinase/akt pathway. Mol Cell.

[79] Potter CJ, Pedraza LG, Xu T (2002). Akt regulates growth by directly phosphorylating Tsc2. Nat Cell Biol.

[80] Hsieh AC (2010). Genetic dissection of the oncogenic mTOR pathway reveals druggable addiction to translational control via 4EBP-eIF4E. Cancer Cell.

[81] She QB (2010). 4E-BP1 is a key effector of the oncogenic activation of the AKT and ERK signaling pathways that integrates their function in tumors. Cancer Cell.

[82] Onda H (1999). Tsc2(+/-) mice develop tumors in multiple sites that express gelsolin and are influenced by genetic background. J Clin Invest.

[83] Kwiatkowski DJ (2002). A mouse model of TSC1 reveals sex-dependent lethality from liver hemangiomas, and up-regulation of p70S6 kinase activity in Tsc1 null cells. Hum Mol Genet.

[84] Ma L (2005). Genetic analysis of Pten and Tsc2 functional interactions in the mouse reveals asymmetrical haploinsufficiency in tumor suppression. Genes Dev.

[85] Nardella C (2008). Aberrant Rheb-mediated mTORC1 activation and Pten haploinsufficiency are cooperative oncogenic events. Genes Dev.

[86] Chen ZJ (2005). Ubiquitin signalling in the NF-kappaB pathway. Nat Cell Biol.

[87] Ozes ON (1999). NF-kappaB activation by tumour necrosis factor requires the Akt serine-threonine kinase. Nature.

[88] Luo JL (2007). Nuclear cytokine-activated IKKalpha controls prostate cancer metastasis by repressing Maspin. Nature.

[89] Chen Z (2005). Crucial role of p53-dependent cellular senescence in suppression of Pten-deficient tumorigenesis. Nature.

[90] Majumder PK (2008). A prostatic intraepithelial neoplasia-dependent p27 Kip1 checkpoint induces senescence and inhibits cell proliferation and cancer progression. Cancer Cell.

[91] Alimonti A (2010). A novel type of cellular senescence that can be enhanced in mouse models and human tumor xenografts to suppress prostate tumorigenesis. J Clin Invest.

[92] Ruggero D (2004). The translation factor eIF-4E promotes tumor formation and cooperates with c-Myc in lymphomagenesis. Nat Med.

[93] Wang H (2010). Skp2 is required for survival of aberrantly proliferating Rb1-deficient cells and for tumorigenesis in Rb1+/- mice. Nat Genet.

[94] Nogueira V (2008). Akt determines replicative senescence and oxidative or oncogenic premature senescence and sensitizes cells to oxidative apoptosis. Cancer Cell.

[95] Jaffe AB, Hall A (2005). Rho GTPases: biochemistry and biology. Annu Rev Cell Dev Biol.

[96] Zhou GL (2003). Akt phosphorylation of serine 21 on Pak1 modulates Nck binding and cell migration. Mol Cell Biol.

[97] Enomoto A (2005). Akt/PKB regulates actin organization and cell motility via Girdin/APE. Dev Cell.

[98] Li J (2005). Phosphorylation of ACAP1 by Akt regulates the stimulation-dependent recycling of integrin beta1 to control cell migration. Dev Cell.

[99] Yoeli-Lerner M (2005). Akt blocks breast cancer cell motility and invasion through the transcription factor NFAT. Mol Cell.

[100] Chin YR, Toker A (2010). The actin-bundling protein palladin is an Akt1-specific substrate that regulates breast cancer cell migration. Mol Cell.

[101] Irie HY (2005). Distinct roles of Akt1 and Akt2 in regulating cell migration and epithelial-mesenchymal transition. J Cell Biol.

[102] Vichalkovski A (2010). PKB/AKT phosphorylation of the transcription factor Twist-1 at Ser42 inhibits p53 activity in response to DNA damage. Oncogene.

[103] Silva BS (2012). TWIST and p-Akt immunoexpression in normal oral epithelium, oral dysplasia and in oral squamous cell carcinoma. Med Oral Patol Oral Cir Bucal.

[104] Liu Z (2012). miR-10b promotes cell invasion through RhoC-AKT signaling pathway by targeting HOXD10 in gastric cancer. Int J Oncol.

[105] Agarwal E, Brattain MG, Chowdhury S (2013). Cell survival and metastasis regulation by Akt signaling in colorectal cancer. Cell Signal.

[106] Wong TS, Gao W, Chan JY (2014). Transcription regulation of E-cadherin by zinc finger E-box binding homeobox proteins in solid tumors. Biomed Res Int.

[107] Arsura M (2003). Transient activation of NF-kappaB through a TAK1/IKK kinase pathway by TGF-beta1 inhibits AP-1/SMAD signaling and apoptosis: implications in liver tumor formation. Oncogene.

[108] McCubrey JA (2017). Roles of GSK-3 and microRNAs on epithelial mesenchymal transition and cancer stem cells. Oncotarget.

[109] Adhikary A (2014). Inhibition of epithelial to mesenchymal transition by E-cadherin up-regulation via repression of slug transcription and inhibition of E-cadherin degradation: dual role of scaffold/matrix attachment region-binding protein 1 (SMAR1) in breast cancer cells. J Biol Chem.

[110] Bolos V (2003). The transcription factor Slug represses E-cadherin expression and induces epithelial to mesenchymal transitions: a comparison with Snail and E47 repressors. J Cell Sci.

[111] Lu P (2011). Extracellular matrix degradation and remodeling in development and disease. Cold Spring Harb Perspect Biol.

[112] Cichon MA, Radisky DC (2014). Extracellular matrix as a contextual determinant of transforming growth factor-beta signaling in epithelial-mesenchymal transition and in cancer. Cell Adh Migr.

[113] Chua HL (2007). NF-kappaB represses E-cadherin expression and enhances epithelial to mesenchymal transition of mammary epithelial cells: potential involvement of ZEB-1 and ZEB-2. Oncogene.

[114] Yoo YA (2011). Sonic hedgehog pathway promotes metastasis and lymphangiogenesis via activation of Akt, EMT, and MMP-9 pathway in gastric cancer. Cancer Res.

[115] Zuo JH (2011). Activation of EGFR promotes squamous carcinoma SCC10A cell migration and invasion via inducing EMT-like phenotype change and MMP-9-mediated degradation of E-cadherin. J Cell Biochem.

[116] Fang D (2007). Phosphorylation of beta-catenin by AKT promotes beta-catenin transcriptional activity. J Biol Chem.

[117] Cha TL (2005). Akt-mediated phosphorylation of EZH2 suppresses methylation of lysine 27 in histone H3. Science.

[118] Li N (2017). AKT-mediated stabilization of histone methyltransferase WHSC1 promotes prostate cancer metastasis. J Clin Invest.

[119] Esteve PO (2011). A methylation and phosphorylation switch between an adjacent lysine and serine determines human DNMT1 stability. Nat Struct Mol Biol.

[120] Huang WC, Chen CC (2005). Akt phosphorylation of p300 at Ser-1834 is essential for its histone acetyltransferase and transcriptional activity. Mol Cell Biol.

[121] Spangle JM, Roberts TM, Zhao JJ (2017). The emerging role of PI3K/AKT-mediated epigenetic regulation in cancer. Biochim Biophys Acta Rev Cancer.

[122] Cheraghchi-Bashi A (2015). A putative biomarker signature for clinically effective AKT inhibition: correlation of in vitro, in vivo and clinical data identifies the importance of modulation of the mTORC1 pathway. Oncotarget.

[123] Pachl F (2013). Characterization of a chemical affinity probe targeting Akt kinases. J Proteome Res.

[124] Erlanson DA (2016). Twenty years on: the impact of fragments on drug discovery. Nat Rev Drug Discov.

[125] De Velasco MA (2016). Efficacy of targeted AKT inhibition in genetically engineered mouse models of PTEN-deficient prostate cancer. Oncotarget.

[126] Smyth LM (2020). Capivasertib, an AKT kinase inhibitor, as monotherapy or in combination with fulvestrant in patients with AKT1 (E17K)-mutant, ER-positive metastatic breast cancer. Clin Cancer Res.

[127] Lin J (2013). Targeting activated Akt with GDC-0068, a novel selective Akt inhibitor that is efficacious in multiple tumor models. Clin Cancer Res.

[128] Spencer A (2014). The novel AKT inhibitor afuresertib shows favorable safety, pharmacokinetics, and clinical activity in multiple myeloma. Blood.

[129] Levy DS, Kahana JA, Kumar R (2009). AKT inhibitor, GSK690693, induces growth inhibition and apoptosis in acute lymphoblastic leukemia cell lines. Blood.

[130] Vicier C (2021). TAKTIC: A prospective, multicentre, uncontrolled, phase IB/II study of LY2780301, a p70S6K/AKT inhibitor, in combination with weekly paclitaxel in HER2-negative advanced breast cancer patients. Eur J Cancer.

[131] Hideshima T (2006). Perifosine, an oral bioactive novel alkylphospholipid, inhibits Akt and induces in vitro and in vivo cytotoxicity in human multiple myeloma cells. Blood.

[132] Becher OJ (2017). A phase I study of single-agent perifosine for recurrent or refractory pediatric CNS and solid tumors. PLoS ONE.

[133] Wu R (2011). Preclinical testing of PI3K/AKT/mTOR signaling inhibitors in a mouse model of ovarian endometrioid adenocarcinoma. Clin Cancer Res.

[134] Liu P (2009). Targeting the phosphoinositide 3-kinase pathway in cancer. Nat Rev Drug Discov.

[135] Holland WS et al. Preclinical modeling of the AKT inhibitor MK2206 and topotecan in small cell lung cancer (SCLC). 2012; 30(15_suppl):e17537.

[136] Al-Saffar NMS (2018). Metabolic biomarkers of response to the AKT inhibitor MK-2206 in pre-clinical models of human colorectal and prostate carcinoma. Br J Cancer.

[137] Lindhurst MJ (2015). Repression of AKT signaling by ARQ 092 in cells and tissues from patients with Proteus syndrome. Sci Rep.

[138] Yu Y (2015). Targeting AKT1-E17K and the PI3K/AKT pathway with an allosteric AKT inhibitor, ARQ 092. PLoS ONE.

[139] Ranieri C (2018). In vitro efficacy of ARQ 092, an allosteric AKT inhibitor, on primary fibroblast cells derived from patients with PIK3CA-related overgrowth spectrum (PROS). Neurogenetics.

[140] Lee JB (2021). Phase 2 study of TAS-117, an allosteric akt inhibitor in advanced solid tumors harboring phosphatidylinositol 3-kinase/v-akt murine thymoma viral oncogene homolog gene mutations. Invest New Drugs.

[141] Politz O (2017). BAY 1125976, a selective allosteric AKT1/2 inhibitor, exhibits high efficacy on AKT signaling-dependent tumor growth in mouse models. Int J Cancer.

[142] Meuillet EJ (2010). Molecular pharmacology and antitumor activity of PHT-427, a novel Akt/phosphatidylinositide-dependent protein kinase 1 pleckstrin homology domain inhibitor. Mol Cancer Ther.

[143] Calleja V (2009). Role of a novel PH-kinase domain interface in PKB/Akt regulation: structural mechanism for allosteric inhibition. PLoS Biol.

[144] Barnett SF (2005). Identification and characterization of pleckstrin-homology-domain-dependent and isoenzyme-specific Akt inhibitors. Biochem J.

[145] Logie L (2007). Characterization of a protein kinase B inhibitor in vitro and in insulin-treated liver cells. Diabetes.

[146] She QB (2008). Breast tumor cells with PI3K mutation or HER2 amplification are selectively addicted to Akt signaling. PLoS ONE.

[147] de Frias M (2009). Akt inhibitors induce apoptosis in chronic lymphocytic leukemia cells. Haematologica.

[148] Zhuang J (2010). Akt is activated in chronic lymphocytic leukemia cells and delivers a pro-survival signal: the therapeutic potential of Akt inhibition. Haematologica.

[149] Arbiser JL (2007). Solenopsin, the alkaloidal component of the fire ant (*Solenopsis invicta*), is a naturally occurring inhibitor of phosphatidylinositol-3-kinase signaling and angiogenesis. Blood.

[150] Arbiser JL (2017). Evidence for biochemical barrier restoration: topical solenopsin analogs improve inflammation and acanthosis in the KC-Tie2 mouse model of psoriasis. Sci Rep.

[151] You I (2020). Discovery of an AKT degrader with prolonged inhibition of downstream signaling. Cell Chem Biol.

[152] Yu X (2022). Discovery of potent, selective, and in vivo efficacious AKT Kinase protein degraders via structure-activity relationship studies. J Med Chem.

[153] Moerke NJ (2007). Small-molecule inhibition of the interaction between the translation initiation factors eIF4E and eIF4G. Cell.

[154] Srivastava RK (2019). Combined mTORC1/mTORC2 inhibition blocks growth and induces catastrophic macropinocytosis in cancer cells. Proc Natl Acad Sci USA.

[155] Wolin E (2019). A phase 2 study of an oral mTORC1/mTORC2 kinase inhibitor (CC-223) for non-pancreatic neuroendocrine tumors with or without carcinoid symptoms. PLoS ONE.

[156] Moore KN (2018). Phase I study of the investigational oral mTORC1/2 inhibitor sapanisertib (TAK-228): tolerability and food effects of a milled formulation in patients with advanced solid tumours. ESMO Open.

[157] Zou Z (2020). mTOR signaling pathway and mTOR inhibitors in cancer: progress and challenges. Cell Biosci.

[158] Sheridan C, Downward J (2013). Inhibiting the RAS-PI3K pathway in cancer therapy. Enzymes.

[159] Zhao H (2020). Targeted inhibition of the E3 Ligase SCF(Skp2/Cks1) has antitumor activity in RB1-deficient human and mouse small-cell lung cancer. Cancer Res.

[160] Zhao H (2013). Skp2 deletion unmasks a p27 safeguard that blocks tumorigenesis in the absence of pRb and p53 tumor suppressors. Cancer Cell.

[161] Rapisarda E (1979). Clinico-statistical evaluation of the anti-plaque efficacy of a new dentifrice. Odontostomatol Implantoprotesi.

[162] Chen Q (2008). Targeting the p27 E3 ligase SCF(Skp2) results in p27- and Skp2-mediated cell-cycle arrest and activation of autophagy. Blood.

[163] Chan CH (2013). Pharmacological inactivation of Skp2 SCF ubiquitin ligase restricts cancer stem cell traits and cancer progression. Cell.

[164] Soucy TA (2009). An inhibitor of NEDD8-activating enzyme as a new approach to treat cancer. Nature.

[165] Vassilev LT (2004). In vivo activation of the p53 pathway by small-molecule antagonists of MDM2. Science.

[166] Ding Q (2013). Discovery of RG7388, a potent and selective p53-MDM2 inhibitor in clinical development. J Med Chem.

[167] Portman N (2020). MDM2 inhibition in combination with endocrine therapy and CDK4/6 inhibition for the treatment of ER-positive breast cancer. Breast Cancer Res.

[168] Endo S (2011). Potent in vitro and in vivo antitumor effects of MDM2 inhibitor nutlin-3 in gastric cancer cells. Cancer Sci.

[169] Konopleva M (2020). MDM2 inhibition: an important step forward in cancer therapy. Leukemia.

[170] Fang Y, Liao G, Yu B (2020). Small-molecule MDM2/X inhibitors and PROTAC degraders for cancer therapy: advances and perspectives. Acta Pharm Sin B.

[171] Tisato V (2017). MDM2/X inhibitors under clinical evaluation: perspectives for the management of hematological malignancies and pediatric cancer. J Hematol Oncol.

[172] Li M (2002). Deubiquitination of p53 by HAUSP is an important pathway for p53 stabilization. Nature.

[173] Li M (2004). A dynamic role of HAUSP in the p53-Mdm2 pathway. Mol Cell.

[174] Colland F (2009). Small-molecule inhibitor of USP7/HAUSP ubiquitin protease stabilizes and activates p53 in cells. Mol Cancer Ther.

