# Correction: Akt: a key transducer in cancer

**DOI:** 10.1186/s12929-023-00900-y

**Published:** 2023-01-25

**Authors:** Pei-Jane Tsai, Yi-Hsin Lai, Rajesh Kumar Manne, Yau-Sheng Tsai, Dos Sarbassov, Hui-Kuan Lin

**Affiliations:** 1 Department of Cancer Biology, Wake Forest University School of Medicine, Winston Salem, NC 27157 USA; 2 Department of Medical Laboratory Science and Biotechnology, College of Medicine, National Cheng Kung University, Tainan, Taiwan; 3 Institute of Basic Medical Sciences, College of Medicine, National Cheng Kung University, Tainan, Taiwan; 4 Institute of Clinical Medicine, College of Medicine, National Cheng Kung University, Tainan, Taiwan; 5 Clinical Medicine Research Center, National Cheng Kung University Hospital, Tainan, Taiwan; 6 Biology Department, School of Sciences and Humanities, and National Laboratory Astana, Nazarbayev University, Nur Sultan City, 010000 Kazakhstan

**Correction: Journal of Biomedical Science (2022) 29:76** 10.1186/s12929-022-00860-9

Following publication of the original article [1], it was found there are some errors in the article:

For the affiliation, one important affiliation is missing for Dr. Yau-Sheng Tsai.

The correct affiliations for Dr. Yau-Sheng Tsai should be:

- 1 Department of Cancer Biology, Wake Forest University School of Medicine, Winston-Salem, NC 27157, USA
- 4 Institute of Clinical Medicine, College of Medicine, National Cheng Kung University, Tainan, Taiwan
- 5 Clinical Medicine Research Center, National Cheng Kung University Hospital, Tainan, Taiwan

For funding section, there is a mistake for “R01CA264020”grant, which needs to be changed to “R01CA256158”. The correct sentence should be:

- This work is supported in part by National Institute of Health (NIH) Grants (R01CA248037 and R01CA256158) to H.K.L and the Grant, AP08857553, from the Ministry of Education and Science of the Republic of Kazakhstan, to D.D.S.

The original paper [1] is updated.
